# A Review of the Involvement of Partners and Family Members in Psychosocial Interventions for Supporting Women at Risk of or Experiencing Perinatal Depression and Anxiety

**DOI:** 10.3390/ijerph18105396

**Published:** 2021-05-18

**Authors:** Maria Noonan, Julie Jomeen, Owen Doody

**Affiliations:** 1 Department of Nursing and Midwifery, Health Research Institute, University of Limerick, V94X5K6 Limerick, Ireland; owen.doody@ul.ie; 2 Faculty of Health, Southern Cross University, Gold Coast Airport, Terminal Dr, Bilinga, QLD 4225, Australia; Julie.Jomeen@scu.edu.au

**Keywords:** systematic search, perinatal depression, perinatal anxiety, psychosocial interventions

## Abstract

A maternal experience of perinatal mental health conditions can have serious short- and long-term consequences for child development and family relationships. Women with perinatal depression and/or anxiety are primarily supported by their partner/spouse and family. The aim of this review was to synthesise data from studies that have examined the inclusion of partners or family members in psychosocial interventions for women at risk of or experiencing perinatal depression and/or anxiety. A systematic search of five databases was conducted to identify literature published between 2010 and 2020. Nine empirical studies met the eligibility criteria and were independently assessed by two authors using the National Heart, Lung and Blood Institute Quality Assessment Tools and data were extracted and narratively synthesised guided by TIDieR (Template for Intervention Description and Replication) checklist. Eligible studies detailed diverse interventions facilitated by a variety of programme facilitators, with no central model of intervention or study outcome measures evident across the studies. All studies except one reported a significant change in maternal depression and anxiety scores. The interventions had limited evaluation of the woman’s, partner’s or family member’s experiences of involvement in the intervention. Further research is required to firmly establish the effectiveness of co-designed interventions to support the sustainable integration of such interventions into routine perinatal mental health services.

## 1. Introduction

In the perinatal period, parents acquire new roles, responsibilities, and knowledge and respond to changes in personal identity, relationships and family dynamics [1,2]. During this transition, parents may experience the spectrum of perinatal mental health ranging from adjustment difficulties to serious mental health conditions [3]. Perinatal depression and anxiety are the most common mental health conditions experienced by both the woman and her partner [4]. An experience of a perinatal mental health condition can influence the long-term health, social, emotional, cognitive and behavioural development of children and impact on family relationships and well-being [5].

The prevalence of depression and anxiety varies across the perinatal period and is dependent on differences within populations, timing of assessments, type of screening instruments, and assessment criteria [4,6]. A meta-analysis reported that perinatal depression affects 12.9% of mothers [7] and estimates ranging from 2.6 to 39% have been reported for perinatal anxiety [8]. In addition, women may simultaneously experience perinatal depression and anxiety; and in the current context of COVID-19, increased prevalence rates of maternal perinatal depression and anxiety have been reported [9].

A family’s mental health is interrelated and the increased demands on fathers’ psychological resources, particularly if they are supporting a partner experiencing perinatal depression and/or anxiety, heightens their vulnerability to stress, anxiety, and depression [2,10]. A meta-analysis of paternal perinatal depression documented prevalence rates of 10.4% [11] and the range increases from 24 to 50% among men whose partners experience perinatal depression [12]. The reported prevalence of paternal anxiety ranges from 3.4% to 25.0% during the antenatal period and 2.4% to 51.0% during the postnatal period [13]. Given the interrelationship between a woman’s perinatal mental health and the health of her partner and child/children, calls have been made for a re-conceptualisation of perinatal mental health as a condition of the family [14,15].

Research has consistently linked relationship factors, including the quality of a relationship with an intimate partner and family support, to the multifactorial aetiology of perinatal depression and anxiety [10,16,17,18,19,20,21]. Instrumental and emotional support from partners and family act protectively to prevent or support recovery from perinatal mental health conditions [1,22], while conflict, poor communication in relationships, and lack of partner and family support can contribute to or exacerbate perinatal depression and anxiety. This may lead to the experience of more severe symptoms of greater duration, impede recovery, increase risk of relapse, and development of additional perinatal mental health conditions [1,22]. Equally, relationship distress may be an outcome of perinatal depression and anxiety [23]. During the perinatal period and particularly in the current context of the COVID-19 pandemic, women have increased dependence on intimate relationships and family support as they experience reduced social interactions with workplaces, the broader community, health care professionals and support networks [9,21,22,24,25,26].

It is important to identify mothers at risk of or experiencing perinatal depression and anxiety and offer prevention and treatment interventions to avert the potential effects of maternal psychological distress on family relationships and the child’s well-being and development. Untreated perinatal depression and anxiety conditions have been associated with adverse child outcomes [27]. Prevention and treatment interventions that include the woman as well as her partner or a significant support person within the family unit may optimise outcomes [1,28,29,30]. The configuration of contemporary families is increasingly diverse across cultures [31] and women may not have a partner or may rely more on the support of family members such as their mother, siblings or other significant persons in their lives. Addressing partner’s and family member’s perinatal mental health literacy and involving partners or family members in prevention and treatment interventions may facilitate earlier maternal help seeking, optimise engagement with interventions and improve psychological and family functioning outcomes [16,29,32,33]. The development of accessible family psychosocial prevention and treatment interventions is an important public health strategy to reduce the impact of perinatal mental health problems on adverse child and parental outcomes [34,35].

There are two approaches to psychosocial interventions for addressing perinatal depression and/or anxiety: (1) preventive programs initiated in pregnancy or early in the postnatal period for women with risk factors for perinatal depression and/or anxiety, and (2) interventions devised to help ameliorate the depressive and/or anxiety symptoms experienced by women with perinatal depression and/or anxiety [36]. This paper presents a review of evidence conducted to explore the involvement of the partner or a family member in interventions developed for the prevention or treatment of women with a history of or who are experiencing perinatal depression and/or anxiety. The findings of this review will inform a discussion of how such interventions are developed and what components of interventions are effective in supporting women at risk of or experiencing depression and/or anxiety. The focus of the discussion is to make recommendations for the development of interventions particularly in the current context of COVID-19, where women have reduced access to services and support structures have changed.

## 2. Materials and Methods

### 2.1. Study Design

The purpose of this review of the literature was to identify studies that detail interventions that include a partner or family member, developed for women who are at risk or currently experiencing perinatal depression and/or anxiety [37,38,39]. This review adhered to the Preferred Reporting Items for Systematic Reviews and Meta-Analyses [40].

#### 2.1.1. Search Strategy and Selection of the Studies

##### Objectives

This review aimed to answer the following questions: What type of interventions that include a partner/family member are available to support women who experience perinatal depression and/or anxiety? How were interventions developed and evaluated? How effective are interventions? The research question for this review is presented in Population, Intervention, Comparison, Outcome (PICO) format in Table 1.

An electronic search was conducted in Cochrane, Cumulative Index to Nursing and Allied Health Literature (CINAHL), Embase, MEDLINE and PsyInfo using key search terms (Table 2).

##### Inclusion and Exclusion Criteria

Included studies had to be based on empirical data (controlled intervention studies or before–after (pre–post) studies with no control group) that explored psychosocial interventions that included a partner/family member, developed for women with a history of or experiencing perinatal depression and/or anxiety. Studies were included that were written in English and published in peer-reviewed journals between 1 January 2010 and 30 April 2020 to ensure currency of interventions. The intervention required a component that included the woman’s partner or family member. “Family member” is defined as one who is biologically related to the mother or a significant-close other with whom she considers being family, but she is not biologically related” [41]. Interventions are categorised as diverse indicated (for women with current symptoms), selective (for women at risk of developing symptoms) and universal (for all, unselected, women) [42]. Only interventions that were diverse or selective were included in this review. Studies were excluded if (a) the study addressed a universal population; (b) the article was a meta-analysis, review paper, qualitative study, case report, case series, scholarly or theoretical paper, editorial, commentary or discussion paper; (c) interventions related to parents of hospitalised premature infants (d); interventions developed for participants with specific diagnoses, such as tocophobia or perinatal loss; (e) the article was in a language other than English.

#### 2.1.2. Data Extraction

**Figure 1:**
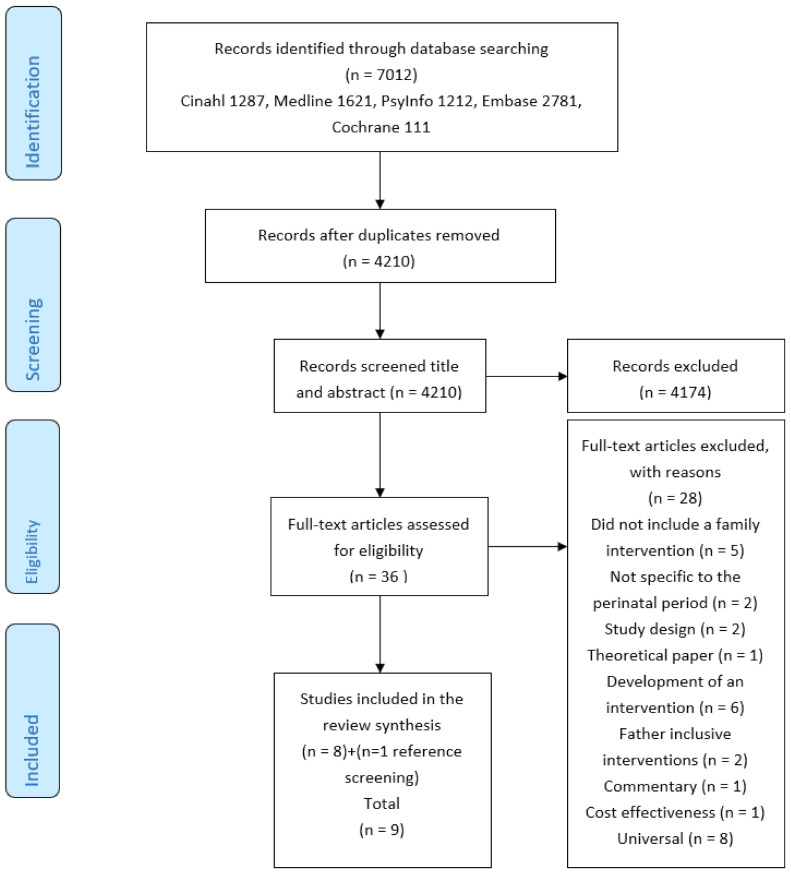
PRISMA 2009 Flow Diagram.

The combined result of electronic searches resulted in the retrieval of 7012 citations which were imported into the reference software ENDNOTE and duplicates removed, leaving 4210 references for review. Titles and abstracts were screened independently by a content expert (MN) against eligibility criteria and discordant opinions were discussed and resolved as they arose (MN and OD). Full texts (n = 36) of potentially eligible studies were retrieved and independently screened for inclusion by two authors (MN and OD). Studies (n = 28) that did not meet the eligibility criteria were excluded and reasons for exclusion were listed on the PRISMA flow diagram (Figure 1). The reference lists of the eight selected studies were reviewed and one further study was identified [43]. Data from nine selected studies were extracted by MN and verified by OD using a standardised data extraction form used to document the following information: purpose, research design, sampling method, study outcome measures, details of interventions, findings, and limitations (Table 3).

#### 2.1.3. Methodological Quality Assessment

The methodological quality of selected studies was independently assessed by two reviewers (MN and OD) using the relevant National Heart, Lung and Blood Institute (NHLBI) Quality Assessment Tools [44] (Tables S1 and S2) and no studies were excluded based on quality. A narrative synthesis of study characteristics, theoretical underpinnings, types of interventions, programme facilitators, the outcome measures relevant to partner or family member, outcome of intervention, and service user involvement in the development of the intervention was conducted. Reporting of details of interventions was guided by the Template for Intervention Description and Replication (TIDieR) checklist and guide [45] (Table 4).

**Table 3:** Data extraction table.

Title, Author/s, Year, Country	Aim/Focus of Paper	Methods	Type of Intervention	Summary of Findings	Appraisal of Included Studies
Marital communication skills training to promote marital satisfaction and psychological health during pregnancy: a couple focused approach (Alipour et al. 2020), Iran [46]	This study was performed to assess the impact of communication skills training on marital satisfaction and levels of depression and anxiety in pregnant women by focusing on the emotional-psychological needs of women during pregnancy.	Study design: Randomised controlled field trial. Sample: 60 pregnant women (control n = 30, intervention n = 30). Study outcome measures: Marital satisfaction and levels of depression and anxiety were re-evaluated before, one and three months after the training course. The ENRICH questionnaire was used to measure marital satisfaction. Subscales of anxiety and depression of the valid GHQ were used to measure maternal depression and anxiety.	A communication skills training package. Training was delivered as lectures, group discussions and role play. The educational subjects of training sessions were focused on the re-establishment of appropriate communication between partners and on understanding the changes and psychological needs of pregnant women and included mindfulness skills.	The level of self- reported depression and anxiety three month after the intervention was lower (*p* = 0.001) and the marital satisfaction (*p* = 0.003) was higher in the intervention group than in the control group. Significant changes in levels of anxiety, depression and marital satisfaction (*p* < 0.001) were reported by participants in the EG at follow up. Results reported a significant inverse correlation between the level of marital satisfaction and anxiety and depression scores before, 2 and 3 months after the intervention.	No outcome data from partners reported. Valid outcome measures. Self-report assessments. No definitive diagnosis of depression and anxiety. Service user involvement in development of the intervention not reported. Short follow-up period (12 weeks).
Video-delivered family therapy for home visited young mothers with perinatal depressive symptoms: Quasi-experimental implementation-effectiveness hybrid trial (Cluxton-Keller et al. 2018), Canada [41]	This 1 year pilot study had the following 2 aims: (1) to explore the feasibility and acceptability of the video-delivered family therapy intervention among home visited families and (2) to explore preliminary impacts of the video-delivered family therapy intervention on maternal depressive symptoms, family functioning, and emotion regulation from baseline to 2 months after the final family therapy session (follow up).	Study design: Pilot wuasi-experimental, implementation-effectiveness hybrid trial. Sample: 13 non-randomised home visited families. Family members: partner/spouse n = 7; Biological family member or close friend n = 6). Historical comparison group of mothers (n= 13). Study outcome measures (baseline, post-intervention, 2 month follow up): The BDI-II (maternal depressive symptoms). The PFS-Family Functioning/Resiliency subscale (family functioning in mothers and their family members). The ERQ (emotion regulation, in mothers and their family members). EPDS scores of mothers in the EG were compared with those of depressed mothers who were previously enrolled in home visiting but refused treatment. Researcher-developed Satisfaction Questionnaire was administered to families post-intervention.	The study intervention was informed by Rathus and Miller (2014), Dialectical Behaviour Therapy (DBT) skills training for adolescents, which includes a multifamily group format and is informed by general systems theory. Families participated in sessions in their homes using cell phones, tablets, and computers equipped with microphones and video cameras. The video-delivered family therapy intervention consisted of 10, 30 min, weekly family therapy sessions that were concurrent with ongoing home visits. It included skills that addressed 3 types of regulation: cognitive, emotion, and behaviour (DBT modules: mindfulness, distress tolerance, emotion regulation, and interpersonal effectiveness).	All families reported high satisfaction with the video-delivered intervention. Mothers demonstrated a statistically significant reduction in depressive symptoms (*p* = 0.001) in comparison to mothers in the historical group. Families demonstrated statistically significant improvements in family functioning (*p* = 0.02) and cognitive reappraisal (*p* = 0.004). The authors found that the significant reduction in maternal depressive symptoms also resulted in an improvement in maternal occupational functioning in that 39% (5/13) of mothers either enrolled in school or were employed for pay by the 2 month follow up. Furthermore, 31% (4/13) of mothers who were either enrolled in school or employed for pay at baseline maintained this status at the 2 month follow up.	Pilot study. Non-randomised small sample size. Historical comparison group. Self-report assessments. Participants were eligible for inclusion in the intervention with EPDS scores of ≥8 because women may underreport symptoms of depression because of associated stigma. Service user involvement in development of the intervention reported in separate publication. The lack of ethnic diversity limits the generalisability of findings. Short follow-up period (8 weeks).
Internet cognitive behavioral therapy for women with postnatal depression: A randomized controlled trial of MumMoodBooster (Milgrom et al. 2016), Australia [47]	Aimed to test the efficacy of a 6-session internet intervention (the MumMoodBooster program, previously evaluated in a feasibility trial) in a sample of postnatal women with a clinical diagnosis of depression.	Study design: Parallel 2-group RCT. Sample: Participants (n = 43) (EG = internet CBT treatment (n = 21) CG = TAU (n = 22)). Study outcome measures: Participants completed the PHQ-9 and DASS-21, ATQ, DAS-7 and PSOC online at enrolment, at weeks 3, 5, 9, and 12 weeks post-enrolment.	The MumMoodBooster program is a CBT intervention, includes a partner website, and was supported by low-intensity non-therapeutic telephone coaching. MumMoodBooster was adapted from Getting Ahead of Postnatal Depression program, which is specifically adapted for the needs of postnatal women. Treatment consisted of six interactive sessions. The programme was delivered using animations, video introductions, case vignettes, audio, video tutorials and self -monitoring activities. Participants received access to a library article on “You and Your Partner” and were able to invite their partner to access the related partner support website with information on PND.	At the end of the study, 79% (15/19) of women who received the internet CBT treatment no longer met diagnostic criteria for depression on the DSM-IV. This contrasted with only 18% (4/22) remission in the TAU. Depression scores on the BDI-II showed a large effect favouring the intervention group (d = 0.83, 95% CI 0.20–1.45). Small to medium effects were found on the PHQ-9 and on measures of anxiety and stress. Adherence to the program—86% (18/21) of users completed all sessions; satisfaction with the program was rated 3.1 out of 4 on average.	No outcome measures identified for partners. Partner use of partner website was not reported. Eligibility screening consisted of an EPDS score of 11 to 23. Women’s diagnostic status was assessed by telephone with the Standardised Clinical Interview for DSM-IV (SCID-IV) and symptom severity with the BDI-II. Small sample size. Self-report assessments. Service user involvement in development of the intervention not reported. The authors reported that women allocated to TAU reported high levels of alternative help seeking and this may have made the detection of true treatment effects relative to TAU more difficult.
Antenatal psychosomatic programming to reduce postpartum depression risk and improve childbirth outcomes: a randomized controlled trial in Spain and France. (Ortiz Collado et al. 2014), Spain and France [48]	The aim of this research was to evaluate the impact of an antenatal programme based on a novel psychosomatic approach to pregnancy and delivery, regarding the risk of PPD and childbirth outcomes in disadvantaged women.	Study design: A multicentre, randomised, controlled trial. Sample: Pregnant women (n = 184) at risk of PPD (EG) (n = 92) and (CG) (n = 92). Study outcome measures: Primary outcome was depressive symptoms (using EPDS) and secondary outcome was preterm childbirth (<37 weeks). Amount of social support received, using the FSSQ 3 stressful events, based on Holmes and Rahe, 1967; and relationship with the partner using DAS, applied separately for women and men. The women completed all the questionnaires, and the men completed questionnaires only concerning the relationship.	The experimental programme used the Tourné psychosomatic approach based on a humanist intervention theory that uses humanistic and cognitive techniques that develop an awareness of feelings and body sensations, their differentiation and their interrelationship. The EG couples participated in 10 small group sessions (two hours) with one telephone conversation between sessions. The group sessions involved work on individual feelings and affective bonds, with specific objectives for the man and the woman in each participating couple.	The experimental intervention using a psychosomatic approach had an impact but did not significantly lower PPD risk. A difference of 11.2% was noted in postpartum percentages of PPD risk (EPDS ≥ 12): 34.3% (24) in EG and 45.5% (27) in CG (*p* = 0.26). The number of depressive symptoms among EG women decreased at T2 (intragroup *p* = 0.01). There was no change in the “relationship with partner” variable in men after childbirth, but it decreased significantly in women who indicated relationship loss or a lack of relationship adjustment after childbirth; the difference was more significant in EG participants. Satisfaction analysis of antenatal programme—no difference is noted for couple communication (3 in the CG and 21 in the EG: *p* = 0.70); improving support in the couple (1 in the CG and 21 in the EG: *p* = 0.10)	Men completed questionnaires only concerning relationships. Ethnic diversity evident with 43% of the participants in the study were immigrants. Pregnant women (n = 184) at risk of PPD (evaluated by validated interview). Self-administered validated questionnaires. Short follow-up period 4 weeks.
Pilot early intervention antenatal group program for pregnant women with anxiety and depression (Thomas et al. 2014), Australia [31]	The aim of the project was to develop and pilot a novel antenatal group program designed to reduce the severity of depression and anxiety symptoms and improve maternal attachment in pregnant women with current or emerging depression and anxiety.	Study design: Pilot antenatal group intervention. Sample: 48 women with antenatal depression or anxiety or deemed at risk of developing PND. Study outcome measures: Women completed pre- and post-treatment measures of depression (CES-D Scale) and EPDS anxiety (STAI) and maternal attachment (MAAS) and the Client Satisfaction Questionnaire. In the final session, participants and their partners who attended the couple session(s) completed an evaluation form aimed to elicit feedback on their experience of the program.	Antenatal group programme based on CBT, IPT and parent–infant interventions theory delivered over 6 sessions. Partners attended 4th and 6th sessions. The program had four core components: (1) several behavioural self-care strategies; (2) psychoeducational (3) interpersonal therapy (IPT) (4) and a parent–infant relationship component. The intervention was delivered through information sharing, group brainstorming, and couple communication activities.	All participants (women and their partners) reported that the program was acceptable and had met their expectations. Significant improvements with moderate to large effect sizes were observed for depression as measured on the CES-D) scale (*p* < 0.001), EPDS (*p* < 0.001), state anxiety (*p* < 0.001) and maternal attachment (*p* = 0.006). Improvements in post-treatment depression scores on the EPDS were maintained at 2 months postpartum. Partners (n = 21) who completed evaluation forms indicated that their attendance had improved their awareness of their partner’s mental health issues and resources available to their family and would recommend the program to other fathers.	Small sample size. No control group. Reliable, self-report measures of anxiety, depression and maternal attachment were employed. This exclusion of women (and their partners) from a diverse range of backgrounds limits the generalisability of findings, particularly in relation to the acceptability of the program. Short follow up period (8 weeks).
MomMoodBooster Web-Based Intervention for Postpartum Depression: Feasibility Trial Results (Danaher et al. 2013), USA and Australia [43]	This pilot study was designed to test the feasibility, acceptability, and potential efficacy of an innovative and interactive guided Web-based intervention for postpartum depression, MomMoodBooster (MMB).	Study design: A feasibility trial of the MMB program Samples: (n= 53) were recruited from two different research sites n = 27, US and n = 26, Australia. Study outcome measures: Assessments occurred at screening/pre-test (corresponding to enrolment), a post-test (3 months following pre-test), and follow up (6 months following pre-test). Women were assessed using the Structured Clinical Interview for DSM-IV Disorders, HRSD, EPDS, PHQ-9, ATQ, BADS, DAS-7, Parenting Sense of Competence (PS Behavioural Self-Efficacy OC) efficacy scale. Website metrics (partners).	The MumMoodBooster program an interactive CBT intervention which includes a partner website and was supported by low-intensity non-therapeutic telephone coaching. The intervention consists of six sequential sessions as follows: (1) Getting Started, (2) Managing Mood, (3) Increasing Pleasant Activities, (4) Managing Negative Thoughts (5) Increasing Positive Thoughts, and (6) Planning for the Future. Programme delivery using animations, video introductions, case vignettes, audio, video tutorials and self -monitoring activities. MMB includes a private peer-based Web forum. Partner support website with information on PND.	A statistical significant decrease (*p* < 0.001) in PHQ-9 scores were reported from pre-test (mean 12.6, SD 4.1) to post-test (mean 5.0, SD 4.4) and the 6 month follow up (mean 4.2, SD 3.9). A statistical significant decrease (*p* < 0.001) in HRSD scores from pre-test (mean 16.9, SD 6.9) to post-test (mean 7.0, SD 5.6) and at 6 month follow up (mean 6.6, SD 6.8) were noted. There was no significant change reported for DAS scores. The mean System Usability Scale score was 84.4 (SD 11.6, range 52.5–100), which translates to a usability grade of “A” for the MMB program.	MMB program was developed using an iterative formative research process that included focus groups and usability testing. Eligibility criteria: EPDS score from 12–20 or a PHQ-9 score from 10–19. Women’s diagnostic status was assessed by phone-administered (SCID and the HRSD No control group. Undertaken across two countries. Validated self-report measures.
Evaluation of a family nursing intervention for distressed pregnant women and their partners: a single group before and after study (Thome and Arnardottir, 2013), Iceland [49]	The aim of this study was to evaluate the clinical effects of an antenatal family nursing intervention for distressed women and their partners on depressive symptoms, anxiety, self-esteem, and dyadic adjustment.	Study design: A single group, before and after, quasi-experimental study. Sample: The pre-test was completed by 61 women and 51 men. Data from the post-test were available for 39 pairs for the EDS, Trait and State Anxiety Inventory (STAI), and RSES and for 35 pairs for the DAS. Study outcome measures: Change in depressive symptoms, anxiety, self-esteem, and dyadic adjustment after the intervention. Four self-report scales: EDS, STAI, RSES and the DAS. In addition, assessment by a genogram was carried out during the first home visit as part of the CFAM.	Family nursing intervention based on the Calgary Family Nursing Model. The model is based on a theoretical foundation involving systems, cybernetics, communication, and change. The intervention model aims to promote mutual cooperation between the family and the nurse to facilitate change or adjustment to a health problem. A hypothesis was constructed for each visit according to suggestions by the authors of the family nursing model (Wright and Leahey 2005) which constituted the focus of the conversation with the couple. The conversation was related to pregnancy and expected parenthood as a transitional period. Partner attendance at first and last of 4 antenatal home visits.	The authors found that couple’s distress was interrelated, and improvement was significant on all indicators after the intervention. Hypothesis 2a,c,e stating ‘Couple’s improvement is interrelated regarding depressive symptoms (EDS), State anxiety (STAI), and the quality of dyadic adjustment (DAS)’ was accepted. Hypothesis 2b,d stating that ‘Couple’s improvement is interrelated regarding Trait anxiety (STAI) and self-esteem (RSES)’ was rejected. Hypothesis 3a–e stating ‘After the intervention there is a significant difference in couple’s depressive symptoms (EDS), Trait and State Anxiety Inventory (STAI), self-esteem (RSES), and the quality of dyadic adjustment (DAS)’ was accepted.	Women attending antenatal care at community health centres who were found to be distressed by midwives were referred to the service. No control group. Validated self-report measures. Small sample size and attrition rate of men noted as a limitation (32% of men participated twice or more, 49% once and 19% never attended. 2/3rds of those who never attended did not complete the post-test). Service user involvement in development of the intervention not reported. No follow up beyond post-test.
Proof of concept: Partner-assisted interpersonal psychotherapy for perinatal depression (Brandon et al. 2012) USA [50]	The aim of this “proof of concept” study was to test safety, acceptability, and feasibility of partner-assisted interpersonal psychotherapy (PA-IPT), an intervention that includes the partner as an active participant throughout treatment.	Study design: Open trial. Sample: Ten couples completed the acute-phase treatment and nine presented for a 6 week follow-up assessment. Study outcome measures: At each session, the woman completed the EPDS and her partner completed the EPDS-P (reporting depressed symptoms partner observed in the woman over the past 7 days). At intake, Session Four (midpoint), Session Eight, and at 6–8 weeks postpartum (or 6–8 weeks following last session if enrolled postpartum), the couple received the HAM-D17 and completed DAS. Four couples attended a focus group held in the last quarter of the project conducted by an independent consultant.	Attachment theory provided the theoretical rationale for PA-IPT which incorporates specific elements borrowed from Emotionally Focused Couple Therapy (EFCT), an evidence-based couple intervention also based upon attachment theory. Couples attended 8 weekly psychotherapy sessions. Three phases of treatment. (1) Accessing the depressive experience from the perspectives of both partners. (2) Role expectations each partner had of self and other, and interactions between them. (3) Consolidation of changes, additional sources of support.	There were significant differences in depressive symptoms (HAM-D17) for the interaction of session by person (*p* < 0.001) and the main effects of session (*p* = 0.001) and person (*p* < 0.001). Women had high levels of depressive symptoms at intake (mean ± standard deviation [SD]: 19.11 ± 6.13) that declined significantly by Session Eight (6.00 ± 4.47) and remained low at the 6 week follow-up evaluation (5.89 ± 2.37). Relationship satisfaction—as measured by the DAS—found no significant main effects of session (*p* = 0.189) or person (*p* = 0.328), and the interaction between session and person was also non-significant (*p* = 0.537). Average scores for women at intake were lower than those of partners (103.10 ± 9.39 versus 105.10 ± 13.68), and scores for women and partners were increased at Session Eight (108.00 ± 16.49 versus 112.70 ± 12.65). All partners reported that they had received personal benefit from the treatment.	Women more than 12 weeks estimated gestational age and less than 12 weeks postpartum were invited to participate if they fulfilled DSM-IV criteria for Major Depressive Disorder and reported moderate symptom severity ≥16 (HAM-D17). Small non-randomised sample size. No control group. Validated self-report questionnaires. Short follow-up period (6–8 weeks).
A randomised control trial for the effectiveness of group interpersonal psychotherapy for postnatal depression (Mulcahy et al. 2010), Australia [20]	The authors aimed to address the unidirectional (one-tailed) hypothesis that an IPT-G intervention is more effective than treatment as usual (TAU) for PPD in a clinical setting drawing from a community sample.	Study design: A randomised control trial. Sample: Mothers with PPD were randomly assigned to IPT-G (n = 23) or TAU (n = 27). Study outcome measures: All participants were asked to complete and return their self-report questionnaires at three time points: week 1 (commencement of IPT-G), week 4 (IPT-G mid-treatment), week 8 (IPT-G end of treatment) and 3 months after the completion of the IPT group. Depression was assessed using the EPDS and the BDI. Marital functioning was measured using the DAS. Social support was assessed using the ISEL. The mother–infant relationship was assessed using the MAI.	The intervention is based on IPT, modified for a group setting (IPT-G). IPT-G consisted of two individual sessions, eight group therapy sessions (2 hours’ duration) and an additional two-hour partner’s evening. This intervention included scope to involve women’s partners in their recovery. Women were given a personalised invitation to give to their partner and a courtesy phone call was made by the group therapist to encourage attendance at a partners evening. The evening was specifically developed for the men only and involved psychoeducation about PPD, with a special emphasis on effective ways to support and respond to their partners.	Comparisons of treatment conditions showed that by end of treatment, both the TAU and IPT-G groups significantly improved in terms of mean depression scores. However, the IPT-G women improved significantly more and had continued improvements at three months post-therapy. Furthermore, women who received IPT-G displayed significant improvement in terms of marital functioning and perceptions of the mother-infant relationship compared to TAU participants.	No outcome measures identified for partners. Diagnosis of postnatal depression based on DSM-IV. Reliance on valid self-report measures. The authors acknowledged that given half of the participants were prescribed antidepressant medication (in both the IPT-G and TAU) it is possible that reduction in symptoms may have been in part due to the antidepressant treatment alone, or antidepressant treatment in combination with another intervention such as the IPT-G. Short follow-up period (12 weeks).

ENRICH, General Health Questionnaire (GHQ); Beck Depression Inventory-second edition (BDI-II); Protective Factors Survey (PFS); Emotion Regulation Questionnaire (ERQ; Edinburgh Postnatal Depression Scale (EPDS); control group (CG); experimental group (EP); cognitive behavioural therapy (CBT); Functional Social Support Questionnaire (FSSQ); dyadic adjustment scale (DAS); Trait and State Anxiety Inventory (STAI); Maternal Antenatal Attachment Scale (MAAS); Centre of Epidemiological Studies Depression Scale (CES-D); Edinburgh Depression Scale (EDS); Rosenberg Self-Esteem Scale (RSES); Structured Clinical Interview for the Diagnosis of Axis I Mental Disorders (SCID-IV, Research version); Hamilton Rating Scale for Depression (HAM-D17); Treatment As Usual (TAU); Depression, Anxiety and Stress Scales—Short Form (DASS-21); Patient Health Questionnaire (PHQ-9); Automatic Thoughts Questionnaire (ATQ); Parenting Sense of Competence Scale (PSOC). Behavioural Activation for Depression Scale (BADS); Hamilton Rating Scale for Depression (HRSD); Rosenberg Self-Esteem Scale (RSES); partner-assisted interpersonal psychotherapy (P-AIPT); Interpersonal Support Evaluation List (ISEL); Maternal Attachment Inventory (MAI); Interpersonal Psychotherapy (IPT).

**Table 4:** TIDier checklist.

		Alipour et al. 2020 [46]	Cluxton-Keller et al. 2018 [41]	Milgrom et al. 2016 [47]	Ortiz Collado et al. 2014 [48]	Thomas et al. 2014 [31]	Danaher et al. 2013 [43]	Thome and Arnardottir, 2013 [49]	Brandon et al. 2012 [50]	Mulcahy et al. 2010 [20]
Brief Name	Provide the name or a phrase that describes the intervention.	Y	Y	Y	Y	Y	Y	Y	Y	Y
Why	Describe any rationale, theory, or goal of the elements essential to the intervention.	Y	Y	Y	Y	Y	Y	Y	Y	Y
What	Materials: Describe any physical or informational materials used in the intervention, including those provided to participants or used in intervention delivery or in training of intervention providers. Provide information on where the materials can be accessed (e.g., online appendix, URL).	Y	?	Y	?	?	Y	?	Y	?
	Procedures: Describe each of the procedures, activities, and/or processes used in the intervention, including any enabling or support activities.	Y	Y	Y	?	Y	Y	Y	Y	Y
Who provided	For each category of intervention provider (e.g., psychologist, nursing assistant), describe their expertise, background and any specific training given.	Y	Y	Y	Y	Y	Y	Y	Y	Y
How	Describe the modes of delivery (e.g., face-to-face or by some other mechanism, such as internet or telephone) of the intervention and whether it was provided individually or in a group.	Y	Y	Y	Y	Y	Y	Y	Y	Y
Where	Describe the type(s) of location(s) where the intervention occurred, including any necessary infrastructure or relevant features.	Y	Y	Y	?	?	Y	Y	?	Y
When and how much	Describe the number of times the intervention was delivered and over what period of time including the number of sessions, their schedule, and their duration, intensity or dose.	Y	Y	Y	Y	Y	Y	Y	Y	Y
Tailoring	If the intervention was planned to be personalised, titrated or adapted, then describe what, why, when, and how.	?	?	?	?	?	?	Y	Y	?
Modifications	If the intervention was modified during the course of the study, describe the changes (what, why, when, and how).	NA	Y	NA	NA	Y	NA	NA	NA	NA
How well	Planned: If intervention adherence or fidelity was assessed, describe how and by whom, and if any strategies were used to maintain or improve fidelity, describe them.	?	Y	Y	Y	Y	Y	?	Y	Y
	Actual: If intervention adherence or fidelity was assessed, describe the extent to which the intervention was delivered as planned.	?	Y	Y	Y	Y	Y	?	Y	Y

*N/A: an item is not applicable for the intervention being described. ‘?’: information about the element is not reported/not sufficiently reported.

## 3. Results

### 3.1. Study Characteristics

The characteristics of the included studies are summarised in Table 3. Studies were conducted in the USA [41,50], Iceland [49], Australia [20,31,47], Iran [46]. Two studies were conducted across two countries Spain/France [48] and USA/Australia [43]. Studies consisted of hospital-based interventions [31,48], community-based interventions; in the home [41,49] or health centres [20,46,50] and internet-based interventions [43,47]. Study designs included randomised controlled trials (RCT) [20,46,47,48] and quasi-experimental and pre-test/post-test studies without a control group [31,41,43,49,50]. Studies examined effects of prenatal interventions for women at risk of developing antenatal depression and/or anxiety [49], at risk of postpartum depression (PPD) or with a current diagnosis of antenatal depression and/or anxiety [31,46], women at risk of postpartum depression (PPD) [48]. Studies reported on interventions for women with self-report perinatal depression [41] a clinical diagnosis of antenatal depression [50] and PPD [20,43,47,50].

### 3.2. Types of Perinatal Interventions

Included studies detailed diverse interventions, with no central model of intervention evident across the studies (Table 3). Alipour et al. [46] developed an antenatal couple-based communication skills training package intervention based on recommendations from two books on communication skills in conjunction with opinions of experts on psychology, psychiatry, and reproductive health conducted over seven two-hour sessions with the couple attending all sessions. Thome and Arnardottir [49] implemented an antenatally family nursing intervention guided by the Calgary Family Nursing Model centred on a theoretical foundation comprising of systems, cybernetics, communication, and change where male partners attended the first and last of four home visits.

Ortiz Collado et al.’s [48] antenatal intervention consisted of the Tourné psychosomatic approach based on a humanist intervention theory that uses humanistic and cognitive techniques that develop an awareness of feelings and body sensations, their differentiation and their interrelationship, delivered over ten weekly sessions lasting two hours attended by the couple. Thomas et al. [31] examined an antenatal group program which comprised of six two-hour sessions held fortnightly, including two sessions (the fourth and sixth sessions) with women and their partners. This programme covered psychoeducation, mood monitoring, adjustment to parenthood, changes in the couple relationship and communication skills.

Cluxton-Kelleher et al.’s [41] (antenatal and postnatal) video-delivered family therapy intervention was informed by Dialectical Behaviour Therapy (DBT) skills training for adolescents and general systems theory and consisted of ten, 30 min, weekly family therapy sessions where family members attended all sessions. Brandon et al. [50] examined the safety, acceptability, and feasibility of partner-assisted interpersonal psychotherapy (PA-IPT) (antenatal and postnatal) consisting of eight weekly psychotherapy sessions attended by the couple.

Milgrom et al. [47] developed MumMoodBooster (MMB), an intervention adapted from the Getting Ahead of Postnatal Depression program, designed for women postnatally. Participants were given a library article on “You and Your Partner” and their partner could access a partner support website (separate log-in process for partners) which had informational resources on PPD. The study built on an earlier feasibility evaluation of MumMoodBooster conducted by Danaher et al. [43].

Mulcahy et al. [20] developed a short-term intervention based on IPA that specifically targets interpersonal relationships and which was modified for a group setting (IPT-G). The IPT-G intervention comprised of two individual sessions, eight group therapy sessions (two hours’ duration), and an additional two-hour evening specifically designed for partners. The evening content explored psychoeducation on PPD, effective strategies for responding to and supporting a partner diagnosed with PPD.

In summary, partners or family members were involved in the intervention at variable levels ranging from partner/family inclusive interventions [31,41,46,48,49,50], psychoeducational interventions [20,43,47] to partner-assisted interventions [50]. Partners or family member was involved in all intervention sessions in four studies [41,46,48,50], two sessions in two studies [31,49], and none in three studies, where partners were referred to a partner-specific support website [43,47] or attended a session specifically designed for partners [20]. Three interventions were facilitated in a group [31,46,48] and one consisted of individual and group sessions [20]. Three interventions were delivered to individual couples, in the home [49], one remotely by video link [41] and the setting is unclear for one study [50]. In two studies, the intervention was delivered through a coach -facilitated website [43,47]. IPT was implemented across two studies, partner-assisted IPT [50] and group IPT [20], and one study had an IPT component [31]. Two interventions were based on CBT [43,47] and one study had a CBT component to the intervention [31]. Communication skills were addressed in six interventions [20,31,41,46,49,50] and two studies had a component on infant parent attachment [31,49]. Overall studies met TIDieR criteria. However, five studies were assessed as not providing adequate details of physical or informational materials used in the intervention (Table 4).

### 3.3. Programme Facilitators

A variety of professional programme facilitators were identified across the interventions ranging from clinical psychologists [31], infant mental health clinicians [31], nurse-midwives [48,49], therapists (researchers) [20,50], specialist in clinical psychology [46], a licensed marriage and family therapist [41], and a telephone coach (three graduate psychology trainees, three clinical psychologists, and one health psychologist who were supported and supervised by two senior psychologists) [43,47].

### 3.4. Outcome Measures Relevant to Partner or Family Member

Outcomes related to partners or family member are detailed in Table 5. Three studies did not report partner outcomes or evaluate partners’ views on the intervention [20,46,47]. The dyadic adjustment scale (DAS) was completed by partners across three studies [48,49,50]. A statistically significant improvement in family functioning (*p* = 0.02) and cognitive reappraisal (*p* = 0.004) at the two month follow up was identified [41]. A Satisfaction Questionnaire developed by the researchers was administered to families post-intervention. All families identified mindfulness skills as the most valuable component of the intervention, followed by emotion regulation (9/13, 69%), distress tolerance (6/13, 46%), and interpersonal effectiveness skills (5/13, 39%) [41]. Participants made no recommendations for changing the intervention.

Ortiz Collado et al.’s [48] reported standard deviation (SD) for men’s dyadic adjustment as 124.80 (18.89) for control group (CG) and 129.10 (10.95) for the experimental group (EG), *p* = 0.32. Global DAS analysis found no significant change between antenatal, 122.68 (17.85) and postnatal scores 129.10 (10.95), *p* = 0.69. Comparative satisfaction analyses found significant differences favouring the EG for some questions including expressing feelings and no significant difference between groups for couple communication (3 in the CG and 21 in the EG: *p* = 0.70) and improving support in the couple (1 in the CG and 21 in the EG: *p* = 0.10) [48].

Findings in Thome and Arnardottir ‘s [49] study accepted the intervention hypothesis stating that ‘After the intervention there is a significant difference in couple’s depressive symptoms (EDS), Trait and State anxiety (STAI), self-esteem (RSES), and the quality of dyadic adjustment (DAS)’. Male participants (n = 10, 25%) who completed both pre-test and post-test reported a significant improvement on the EDS as their scores dropped between 4 and 10 points [49].

In Brandon et al.’s [50] study, partners were more attuned to the woman’s symptoms of PND at the end of the intervention. While partners reported no significant changes on DAS, they indicated that they experienced personal benefit from attending the intervention [50].

A general evaluation of the programme from the partner’s perspective was examined by Thomas et al. [31] and partners (n = 21) indicated that their attendance had enhanced awareness of their partner’s perinatal mental health and resources accessible to their family and would recommend the program to other fathers. Danaher et al. [43] used analytic tools to track website metrics including patterns of visits to website and found that 34% (18/53) of partners accessed the MMB partner support website. Overall, in studies that examined outcomes related to partner or family member, findings suggest that some partner’s/family members benefited from the intervention in terms of improved family functioning, relationships and perinatal mental health literacy.

### 3.5. Outcome of Interventions (Maternal Outcomes, Relationship Outcomes)

#### 3.5.1. Maternal Outcomes

All studies except one [48] reported significant changes in maternal depression and/or anxiety scores (Table 5). Ortiz Collado et al. [48] identified no significant difference in maternal PPD scores on the EPDS between the EG and CG for the antenatal intervention. Women in the EG had a significant decrease in the number of self-reported postnatal depressive symptoms (*p* = 0.01) when compared with the prenatal test.

#### 3.5.2. Relationship Outcomes

Across five studies, women reported significant improvements in outcomes that evaluated relationships with their partner or family functioning. Alipour et al. [46] reported that for pregnant women, levels of marital satisfaction increased significantly in the intervention group (*p* < 0.05) one and three months post-intervention. Thome and Arnardottir [49] found improvement in the couples score for DAS (*p* = 0.001) post-intervention. Similarly, Mulcahy et al. [20] reported significantly better marital relationships in the IPT-G group in comparison to participants in the CG, with effects of the intervention maintained at three months follow up. Furthermore, Cluxton-Kelleher et al. [41] reported statistically significant improvements in family functioning (*p* = 0.02). In contrast, three studies did not find an improvement in relationships. Milgrom et al. [47] found that the intervention had no statistically significant effect on partner relationship scores (DAS-7) for women, although its impact on the parenting self-efficacy measure (PSOC) constituted a medium effect size [47]. Similarly, in Brandon et al.’s [50] study, women reported no significant changes on DAS. Ortiz Collado et al. [48] identified no change in the “relationship with partner” variable in men after childbirth, whereas women in the EG reported a greater lack of adjustment in their relationship with the baby’s father postpartum. The authors hypothesised that the separation rate and other relationship problems may have been underreported in the CG.

### 3.6. Involvement of the Woman, Partner, or Family Member in the Development of the Intervention

Many studies [20,41,46,48,49,50] do not state whether women, partners, or their family members were engaged in the design and development of the intervention. Danaher et al. [43] and Milgrom et al. [47] used an iterative formative research process (focus groups and usability testing) to develop the MMB programme. Thomas et al. [31] reported that the content of the draft program was evaluated and refined by consumers who were requested to reflect upon what information and resources that they would have found beneficial to have in their pregnancy. The consumers were past patients and had received psychological therapy for PPD. The initial programme consisted of one partner session, which was increased to two partner sessions based on feedback from participants and partners. We acknowledge that service user involvement in the development of interventions may be reported in separate linked publications, a review of which was beyond the scope of this review.

## 4. Discussion

This review identified nine interventions developed for women experiencing or at risk of perinatal depression and/or anxiety that included a partner/family member. Interventions were highly heterogeneous, with variation in intervention type, timing of delivery, target populations, level of inclusion of partner/family member in the intervention, and outcome measures, making comparison across studies almost impossible. Some studies did not evaluate outcome measures related to the partner or family member. While in the main, the studies reviewed report perceived positive outcomes, with reductions in self-reported levels of maternal perinatal depression and anxiety. There was limited evidence of involvement of the woman or the family in the development of the intervention coupled with limited evaluation of partner’s or family member’s experiences of involvement in the intervention. This is significant as partners and family members can influence and support improved health outcomes [51]. The involvement of the family is essential in providing practical and emotional support to the woman [42] and can contribute to normalising support seeking within families and communities [52].

The findings of this review suggest that technology-enhanced programmes maybe a feasible and acceptable means of delivering interventions [41,43,47]. Web-based perinatal mental health psychological interventions, with therapist or coach guidance, represent a rapidly emerging and promising approach due to their adaptability, non-intrusive nature and the increasingly global access to online platforms which can extend the reach of effective interventions [53,54]. Technology offers one solution in which women can have more direct control over their treatment schedule and has the potential to overcome known and current barriers to accessing treatment including in the context of COVID-19 such as perceived stigma, unequal availability, and difficulties in assessing in-person clinic-based therapies and because of logistical challenges associated with childcare [47]. However, in considering tele/digital health cultural differences, inequalities in technology access and digital literacy must be considered as these may make it more challenging in comparison to face-to-face consultations and in-person treatment may be a necessity, particularly for those needing intensive perinatal mental health interventions, care and support [55].

The literature acknowledges that within individual diagnostic constructs such as perinatal depression and anxiety, there are different phenotypes, potentially requiring diverse and culturally responsive interventions and services [33]. Mulcahy et al. [20] identified the specific therapeutic benefits of peer support through sharing experiences with other women while participating in group IPT sessions including the reduction in isolation and feelings of loneliness, as well as normalisation of perinatal mental health problems, through the creation of a sense of universality of concerns. However, women have reported contrasting perceptions and experiences of group CBT for PPD [56]. A challenge going forward is how to identify women and partners that are most likely to benefit from psychosocial interventions in a group environment [56] and recreate the benefits of group support and interaction in an online setting.

The value of service user input in interventions is now widely accepted and many selected studies did not report the inclusion of service users in the development of the intervention and we acknowledge that this may be reported in other publications related to the intervention. The literature recognises that intervention development processes are rarely reported and that there is little information provided about the extinct of service user involvement in intervention design [57]. The UK Medical Research Council’s guidance [58] on the development and evaluation of complex interventions underscores the importance of preliminary design development, which explores the intervention from the perspective of the target population to optimise acceptability of the intervention. Male partners process information differently than women and value practical guidance and it is important to take into consideration fathers’ perspectives when designing an intervention that includes partners. Perinatal mental health teams providing services need to enhance their understanding in relation to the resource’s fathers require to support their own and their partners’ perinatal mental health [59]. Involving potential recipients of an intervention at the design phase may be especially important when the intervention aims to realise behavioural, as opposed to biochemical change [57]. Notably absence from evaluation of interventions was the woman’s perspective of having her partner/family member involved. The woman should be central to the design and delivery of interventions and facilitating service user involvement will invariably result in more appropriate interventions of a higher quality with increased service user satisfaction.

A woman’s social network including her partner and family usually provides primary or additional support to that provided by perinatal mental health services [59]. Partners are identified as the main support system and have a central role to play in the recognition of the woman’s perinatal mental health status. However, many men feel excluded and unclear about their role which suggests a diminished appreciation of the father’s role in supporting maternal perinatal mental health [59]. One study [50] that screened men’s mental health status pre-intervention did not have a component to address men’s mental health instead men with undiagnosed psychiatric conditions identified on screening were excluded from the study and referred to community mental health providers. A second study [49] found that 25% of men that completed both pre- and post-mental health assessments reported a clinically significant improvement on the EDS as their scores dropped between 4 and 10 points. Enduring beliefs concerning masculinity and fatherhood deter men from seeking support for their perinatal mental well-being [16]. Concurrently screening partners of eligible women included in psychosocial interventions may offer an opportunity to link men who experience perinatal depression and/or anxiety to services [59]. One study in this review [49] found that the couple’s improvement regarding depressive symptoms, state anxiety and dyadic adjustment was interrelated, suggesting that interventions that address both maternal and paternal mental health may lead to improved outcomes for the family.

Only one study included family members in the intervention [41]. Lower rates of postnatal depression have been reported by women who identified higher perceived support from their own mother [16]. The inclusion of family members in psychosocial interventions offers a holistic approach that has the potential to provide broader benefits to family relationships, dynamics, systems, and child outcomes and may be more effective than partner support alone [28,30]. There is now an increasing emphasis in policy in some countries to include and support families of women who experience perinatal mental health difficulties. However, limited research has focused on the support needs of family members [16]. In a study undertaken in the UK, women, their partners, and wider family members suggested that families were marginalised by services and families desired more family-oriented or holistic perinatal mental services [16]. However, for a variety of reasons, including complex interpersonal dynamics, not all women would want or benefit from family involvement. Women from all backgrounds including ethnic minority groups, may not disclose their psychological distress or attendance for treatment to their families because of perceived stigma around perinatal mental health within their wider family and social networks [16]. Furthermore, time alone with a therapist may be vital for women who experience coercive and abusive relationships [16]. Only three studies had exclusion criteria related to current domestic or family violence [41,48,50] and one study reported that a couple were disqualified after partner violence (female upon male) was revealed [50]. Furthermore, Brandon et al. [50] developed their intervention which focused on women with perinatal depression and anxiety who required increased partner support and not for women who reported relationship discord. They argued that serious relationship conflict contraindicates the inclusion of a partner in an intervention as it may contribute to a dysfunctional balance of power in the relationship, particularly when the woman is seeking support for psychological distress [50].

Authors suggest that health care professionals need to identify the optimal level of partner and family involvement to ensure interventions are prioritised to the needs of women and babies, thus ensuring women themselves are not marginalised [16]. Health care professionals, however, may not have the appropriate resources or training to identify the diverse and individual needs of women, partners and family members, particularly where interpersonal relationships are strained [16]. In addition, many health care professionals would not see women in a context where the couple or family dynamics are obvious. This would potentially need a different kind of interaction with women beyond screening their perinatal mental health. Family members have also expressed ambivalence about being involved in treatment and developers of interventions need to safeguard against the possibility of making assumptions regarding what might be most helpful for families or how the intervention might best work [16].

### 4.1. Strengths and Limitations

There is difficulty in forming firm conclusions from this review as the format and contents of interventions were widely varied within each approach, the international nature of the studies creates ecological variance, and women and partners were screened and assessed using different tools, criteria, and largely at different times. We were unable to determine whether the partner/family component which varied from partner/family inclusive to partner-assisted interventions led to the reported improvements in perinatal mental health. Improvements in relationships were evident even when there were no concurrent partner sessions [20,43,47]. Furthermore, while women were excluded in three studies if they were receiving concurrent treatment [43,49,50], it was noted in one study that women were availing of concurrent treatment [20], in a second study that women were not excluded if receiving concurrent treatment [31] and the possibility of concurrent treatment was not addressed in other studies [41,47,48] (Tables S1 and S2 file).

The reliability of included studies may have been further strengthened using a combination of self-report measures and diagnostic measurement methods to determine study eligibility and to evaluate intervention outcomes [20,43]. The included studies in the main had small sample sizes [20,41,46,47,50], clear baseline characteristics and adequate presentation and analysis of findings and authors had accounted for attrition rates in interventions [20,41,46,47,48,49,50]. Four studies did not include an active control group [31,43,49,50] and one study used a historical comparison group [41].

Some studies did not evaluate the intervention from the partner/family member perspective [20,46] and overall studies did not evaluate the woman’s perspective of having a partner or family member involved.

Only five databases were searched and one author screened the title and abstract of retrieved articles, print and grey literature was not searched, and published data were restricted to English language. Therefore, there is a possibility of language bias and missing relevant published and unpublished studies. Despite these limitations, the narrative synthesis of findings from eligible studies provides an overview of the types of interventions developed for women that included a partner or family member component and guides recommendations for future developments in this area.

### 4.2. Recommendations

The inclusion of a partner/family member in the prevention/treatment intervention offers a holistic approach to caring for women who are experiencing perinatal depression or perinatal depression and anxiety, yet the evidence remains underdeveloped. Service user involvement (the woman, partner, family member) in the design and development of the intervention may strengthen interventions as would greater consideration to appropriate theoretical and evidence-based underpinnings. Interventions need to consider including an element that addresses the burden placed on the partner/family member. A couples’ perinatal mental health co-morbidity may impact on the success of the intervention as men experiencing perinatal mental health needs may not be able to provide the support intended by the intervention. Therefore, interventions need to plan for the possibility of the partner experiencing depression and/or anxiety during the intervention. Intervention eligibility criteria that screen for family violence which may affect either partner is recommended. Relationships where there is existing conflict or where relationship distress emerges as an outcome of perinatal depression and anxiety may require a different intervention format and support structure to that where relationships are identified as strong at the beginning of the intervention. Current interventions included either a partner or family member and future interventions need to consider components that actively include both the woman’s partner and her family members where appropriate. Further research is required to investigate whether interventions address the needs of the woman, partner and family member and how such interventions influence the mother’s perinatal mental health and family relationships. Most interventions did not target depression and anxiety concurrently and addressing this will be important going forward as evidence suggests that anxiety is common. Measurement of longer-term outcomes including economic outcomes are important for future evaluation as women with perinatal depression may experience relapse and in order to establish whether this reduction changes or remains stable over time. Finally, the addition of a component on Public and patient involvement in research (PPI) to the TiDieR checklist may be warranted.

## 5. Conclusions

This review identified the types of interventions available to women at risk of or experiencing perinatal depression and/or anxiety that included a partner/family member. Further research is required to firmly establish the effectiveness of co-designed interventions adapted to different cultures in order to support the sustainable integration of such interventions into routine perinatal mental health services.

## Figures and Tables

**Table 1:** PICO.

Population	Women at risk of or experiencing perinatal depression or perinatal depression and anxiety
Intervention	Psychosocial interventions that include a partner/family member
Comparison	Standard/usual care
Outcome	Types of interventions, the outcome of interventions for the woman, partner, family member

**Table 2:** Search terms.

S1
Perinatal OR peri-natal OR Peripartum OR partum OR postpartum OR pre-natal OR prenatal OR puerperal OR puerperium OR postnatal OR intrapartum OR childbirth OR childbearing OR antenatal OR pregnan* OR trimester* OR birth* OR gestation
S2
depressive disorder OR depressive disorder major OR mood disorders OR dysthymic disorder OR depression OR depressive OR depressed OR dysthymia OR dysthymic OR affective symptoms OR affective disorder OR affective disorders OR anxiety OR anxiety disorder OR anxio* OR panic OR obsessi* OR compulsi* OR OCD OR GAD
S3
Famil* OR Significant other OR Spouse OR Husband* OR Wife OR Wives OR Partner*
S4
intervention OR therap* OR treatment* OR train* OR educat* OR program* OR psychosocial* OR psychological OR counsel* OR support OR psychotherap* OR coping OR cognitive behavio$ral OR CBT
S1 AND S2 AND S3 AND S4

**Table 5:** Outcomes.

Author/s	Maternal Outcomes	Outcome Measures Relevant to Partner or Family Member
Alipour et al., 2020 [46]	Alipour et al. [46] reported that for pregnant women, levels of depression and/or anxiety decreased significantly in the intervention group (*p* < 0.05) at one and three months postintervention.	Not reported.
Cluxton-Keller et al., 2018 [41]	Significant reductions were reported in maternal depressive symptoms (*p* = 0.001) at the 2 month follow up, and statistically significant improvements in cognitive reappraisal (*p* = 0.004) at the 2 month follow up.	A statistically significant improvement in family functioning (*p* = 0.02) and cognitive reappraisal (*p* = 0.004) was reported at the 2 month follow up. All families identified mindfulness skills as the most valuable component of the intervention, followed by emotion regulation (9/13, 69%), distress tolerance (6/13, 46%), and interpersonal effectiveness skills (5/13, 39%).
Milgrom et al., 2016 [47]	At the end of the study, 79% (15/19) of women who received the internet CBT treatment no longer met diagnostic criteria for depression on the DSM-IV. This contrasted with only 18% (4/22) remission in the TAU condition. Depression scores on the BDI-II showed a large effect favouring the intervention group (d = 0.83, 95% CI 0.20–1.45). Small to medium effects were found on the PHQ-9 and on measures of anxiety and stress.	Not reported.
Ortiz Collado et al., 2014 [48]	No significant difference in maternal PPD scores on the EPDS between the experimental group (EG) and control groups (CG) for the antenatal intervention identified. Women in the EG had a significant decrease in the number of self-reported postnatal depressive symptoms (*p* = 0.01) when compared with the prenatal test.	SD for men’s dyadic adjustment on DAS was reported as 124.80 (18.89) for CG and 129.10 (10.95) for the EG, *p* = 0.32. Gobal DAS analysis found no significant change between antenatal 122.68 (17.85) and postnatal scores 129. 10 (10.95), *p* = 0.69. Comparative satisfaction analyses found significant differences favoring the EG for some questions including expressing feelings and no significant difference between groups for couple communication (3 in the CG and 21 in the EG: *p* = 0.70) and improving support in the couple (1 in the CG and 21 in the EG: *p* = 0.10).
Thomas et al., 2014 [31]	A significant reduction was reported in participants’ level of depression as measured on the CES-D (*p* < 0.001), state anxiety (*p* < 0.001) and significant improvement in maternal attachment (*p* = 0.006) post-treatment. Improvements in post-treatment depression scores on the EPDS were maintained at 2 months postpartum.	Partners (n = 21) who completed a general evaluation of the programme indicated that their attendance had improved their awareness of their partner’s perinatal mental health and resources available to their family and would recommend the program to other fathers [31].
Danaher et al., 2013 [43]	A statistical significant decrease was reported (*p* < 0.001) in PHQ-9 scores from pre-test (mean 12.6, SD 4.1) to post-test (mean 5.0, SD 4.4) and the 6 month follow up (mean 4.2, SD 3.9). A statistical significant decrease (*p* < 0.001) in HRSD scores from pre-test (mean 16.9, SD 6.9) to post-test (mean 7.0, SD 5.6) and at 6 month follow up (mean 6.6, SD 6.8) were noted.	Danaher et al. [43] used analytic tools to track website metrics including patterns of visits to website and found that 34% (18/53) of partners accessed the MMB partner support web-site.
Thome and Arnardottir, 2013 [49]	Thome and Arnardottir [49] found that the couple’s distress was interrelated and reported significant improvement in the couples score for the EDS (0.001), and STAI state (0.001) post-intervention.	The couple completed the EDS, STAI, RSES, and the DAS. The findings of the study accepted the intervention hypothesis stating that ‘After the intervention there is a significant difference in couple’s depressive symptoms (EDS), Trait and State Anxiety Inventory (STAI), self-esteem (RSES), and the quality of dyadic adjustment (DAS)’. Male participants (n = 10, 25%) reported a significant improvement on the EDS as their scores dropped between 4 and 10 points.
Brandon et al., 2012 [50]	Women reported high levels of depressive symptoms at baseline (mean ± standard deviation [SD]:19.11 ± 6.13) that declined significantly by session eight (6.00 ± 4.47) and remained low at follow up (5.89 ± 2.37).	Partners completed the HAM-D17 and DAS. While partners reported no significant changes on DAS, they indicated that they experienced personal benefit from attending the intervention. Partners rated the intensity of symptoms of maternal depression lower at commencement of the intervention (EPDS-P scores mean ± SD = 13.80 ± 3.36) and by session eight, partner ratings demonstrated more agreement with women’s ratings (6.10 ± 4.48) [50].
Mulcahy et al., 2010 [20]	Participants in the IPT-G reported an overall decrease in depression scores, sustained decrease in symptoms by three months follow up, a higher number of women who met recovery criteria (IPT-G 69.6% vs. TAU 33.3%), and improvement in interpersonal functioning. Participants reported significantly better marital relationships in the IPT-G group in comparison to participants in the CG, with effects of the intervention were maintained at three months follow up.	Not reported.

## Supplementary Materials

The following are available online at https://www.mdpi.com/article/10.3390/ijerph18105396/s1, Table S1: Quality Assessment of Controlled Intervention Studies, Table S2: Quality Assessment Tool for Before-After (Pre-Post) Studies with No Control Group.
