# Supplementary material for: A Review of the Involvement of Partners and Family Members in Psychosocial Interventions for Supporting Women at Risk of or Experiencing Perinatal Depression and Anxiety
Source: Int J Environ Res Public Health. 2021 May 18;18(10):5396. doi: 10.3390/ijerph18105396 (PMC8158393; doi:10.3390/ijerph18105396)
Supplement: Supplementary file 1 [file ijerph-18-05396-s001.zip › ijerph-1201733-supplementary.pdf]

Supplementary file: Quality Appraisal

Table S1. Quality Assessment of Controlled Intervention Studies.

Appraisal questions	Alipour et al 2020 [49]	Milgrom et al 2016 [48]	Ortiz Collado et al 2014 [50]	Mulcahy et al 2010 [20]
1. Was the study described as randomized, a randomized trial, a randomized clinical trial, or an RCT?	Yes	Yes	Yes	Yes
2. Was the method of randomization adequate (i.e., use of randomly generated assignment)?	Yes	Yes	Yes	Yes
3. Was the treatment allocation concealed (so that assignments could not be predicted)?	Yes	Yes	Yes	Yes
4. Were study participants and providers blinded to treatment group assignment?	No	No	No	No
5. Were the people assessing the outcomes blinded to the participants' group assignments?	Yes	Yes	CD	Yes
6. Were the groups similar at baseline on important characteristics that could affect outcomes (e.g., demographics, risk factors, co-morbid conditions)?	Yes	Yes	Yes	Yes
7. Was the overall drop-out rate from the study at endpoint 20% or lower of the number allocated to treatment?	Yes	Yes	No	Yes
8. Was the differential drop-out rate (between treatment groups) at endpoint 15 percentage points or lower?	Yes	Yes	No	Yes
9. Was there high adherence to the intervention protocols for each treatment group?	Yes	Yes	Yes	Yes
10. Were other interventions avoided or similar in the groups (e.g., similar background treatments)?	Yes	CD	CD	No.
11. Were outcomes assessed using valid and reliable measures, implemented consistently across all study participants?	Yes	Yes	Yes	Yes
12. Did the authors report that the sample size was sufficiently large to be able to detect a difference in the main outcome between groups with at least 80% power?	Yes	No.	No.	Yes.
13. Were outcomes reported or subgroups analyzed prespecified (i.e., identified before analyses were conducted)?	Yes	Yes	Yes	Yes
14. Were all randomized participants analyzed in the group to which they were originally assigned, i.e., did they use an intention-to-treat analysis?	CD	Yes	Yes	No.

Keys Y (Yes), N (No), Other (CD, cannot determine; NA, not applicable; NR, not reported)

https://www.nhlbi.nih.gov/health-topics/study-quality-assessment-tools

Table S2. Quality Assessment Tool for Before-After (Pre-Post) Studies with No Control Group

Appraisal questions	Cluxton-Keller et al 2018 [41]	Thomas et al 2014 [31]	Danaher et al 2013 [43]	Thome & Arnardot-tir, 2013 [47]	Brandon et al 2012 [46]
1. Was the study question or objective clearly stated?	Yes	Yes	Yes	Yes	Yes
2. Were eligibility/selection criteria for the study population pre-specified and clearly described?	Yes	Yes	Yes	Yes	Yes
3. Were the participants in the study representative of those who would be eligible for the test/service/intervention in the general or clinical population of interest?	Yes	Yes	Yes	Yes	Yes
4. Were all eligible participants that met the pre-specified entry criteria enrolled?	Yes	Yes	Yes	Yes	Yes
5. Was the sample size sufficiently large to provide confidence in the findings?	CD	CD	CD	Yes.	CD
6. Was the test/service/intervention clearly described and delivered consistently across the study population?	Yes	Yes	Yes	Yes	Yes
7. Were the outcome measures prespecified, clearly defined, valid, reliable, and assessed consistently across all study participants?	Yes	Yes	Yes	Yes	Yes
8. Were the people assessing the outcomes blinded to the participants' exposures/interventions?	CD	No	No	CD	CD
9. Was the loss to follow-up after baseline 20% or less? Were those lost to follow-up accounted for in the analysis?	Yes	CD	Yes	No	Yes
10. Did the statistical methods examine changes in outcome measures from before to after the intervention? Were statistical tests done that provided p values for the pre-to-post changes?	Yes	Yes	Yes	Yes	Yes

11. Were outcome measures of interest taken multiple times before the intervention and multiple times after the intervention (i.e., did they use an interrupted time-series design)?	Yes.	Yes	Yes	Pre and post test	Yes
12. If the intervention was conducted at a group level (e.g., a whole hospital, a community, etc.) did the statistical analysis take into account the use of individual-level data to determine effects at the group level?	NA	NA	NA	NA	NA

Keys Y (Yes), N (No), Other (CD, cannot determine; NA, not applicable; NR, not reported)

https://www.nhlbi.nih.gov/health-topics/study-quality-assessment-tools
